# Supplementary material for: Guidance to best tools and practices for systematic reviews
Source: J Pediatr Rehabil Med. 2023 Jun 7;16(2):241–73. doi: 10.3233/PRM-230019 (PMC10258887; doi:10.3233/PRM-230019)
Supplement: Supplementary File 2B [file prm-16-prm230019-s002.pdf]

Supplemental File 2B: Practical scheme for distinguishing research evidence

Application of this scheme (Figure SF2B) facilitates identification of the main types of research available for inclusion in evidence syntheses. Note this same diagram is presented and briefly described in Part 2 of the main text.

An initial distinction is made between primary studies, which are reports of original research, and secondary studies, which are more commonly referred to as evidence syntheses. These include traditional systematic reviews defined by the topics they assess as well as other types of evidence syntheses recognized by Cochrane and JBI (See Tables 2.1 and 2.2 in the main text). Notably, while evidence summaries in traditional systematic reviews are based exclusively on data reported by primary studies (eg, randomized controlled trials, non-randomized studies of interventions [NRSI]), other types of evidence syntheses may consider data only from secondary studies (eg, overviews or umbrella reviews) or from both primary and secondary studies (eg, scoping reviews).

Details about the different types of secondary studies are presented in Part 2 of the main text. Guidance for making distinctions between the different types of primary studies represented in the Practical Scheme (Figure SF2B) is offered below.

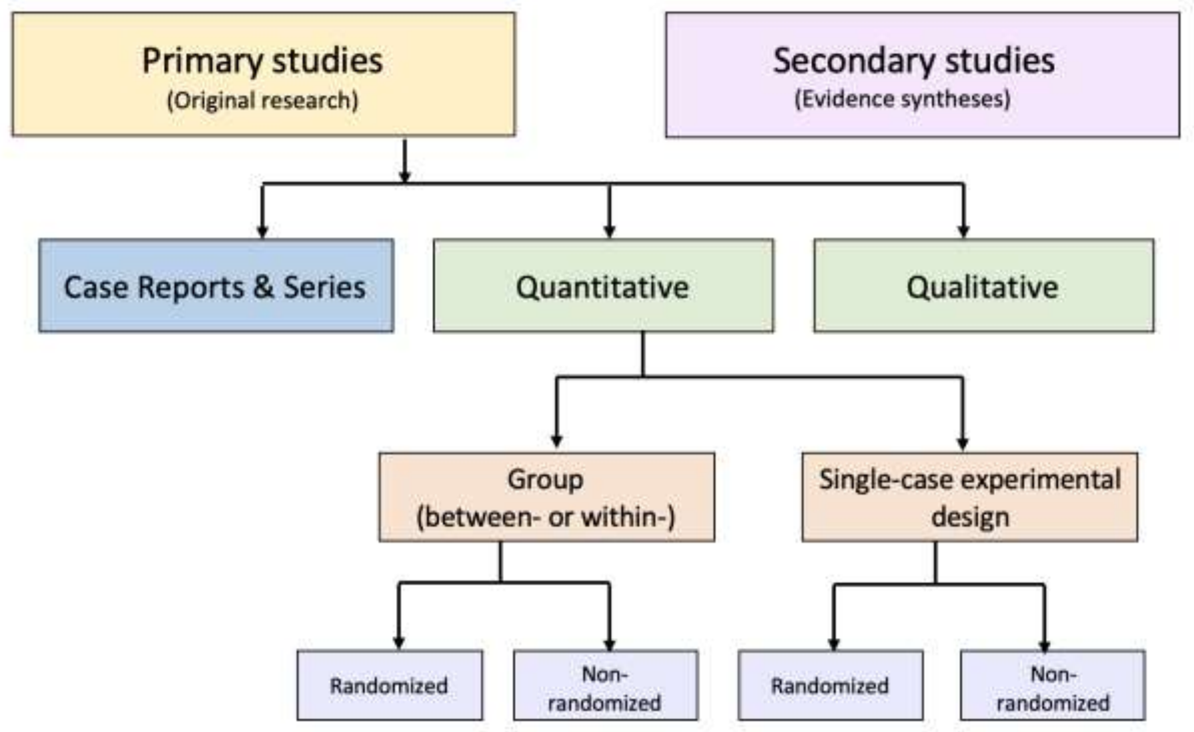

FIGURE SF2B: Distinguishing types of research evidence

Kolaski, K., Logan, L., & Ioannidis, J. P. A. (2023). Guidance to best tools and practices for systematic reviews. Journal of Pediatric Rehabilitation Medicine. DOI:10.3233/PRM-230019.

Distinctions based on the type of data reported

Primary studies typically report either quantitative data or qualitative data. Quantitative data are expressed numerically and analyzed statistically; they are collected from experiments and tests, metrics, databases, and surveys. Such data are commonly reported in healthcare research including studies of intervention effectiveness, satisfaction with care, the incidence, prevalence, and etiology of diseases, and the properties of measurement tools.[1] Qualitative data are descriptive (eg, concepts, meanings, words, etc.) rather than numerical and are collected through interviews, observations, and textual analyses. Qualitative research studies in healthcare investigate the impact of illnesses and interventions and explore the experiences, attitudes, beliefs, and perspectives of patients, caregivers, and clinicians.[2] Qualitative systematic reviews synthesize this data using meta-aggregation[2] or an interpretative approach (eg, meta-ethnography, critical interpretative synthesis, realist synthesis).[3]

Distinctions between primary studies reporting quantitative data

Group and single case

These two broadly defined approaches may attempt to establish causal relationships or describe associations.[4] In group research, data collected from groups of individuals are analyzed and allow for testing the effectiveness of treatments at the group level. "Between group" designs are typical of clinical research in medicine. These studies compare participants that have different exposures (eg, control *versus* experimental) or that differ on some feature (eg, gender, disease risk factor, test measurement or score).[5] Less commonly, studies of groups utilize a "within group" design (also referred to as "within-subjects"). Such studies collect data from groups of participants exposed to the same condition at various times (eg, before/after, or with repeated exposures).

Single case experimental designs are also known as single-subject, N-of-1, or small-n designs. These are also characterized by repeated measurements over time in participants with the same exposures; however, in contrast to group design research, the individual case serves as the unit of analysis. This may be one person or an entity such as a classroom or an organization[6]; for this reason, we prefer use of "single case experimental design" (SCED) to describe these studies. SCEDs typically involve numerous repeated measurements along with multiple methods for ensuring accuracy and fidelity of the data.[7] Confidence in the validity of the data from individuals or entities may be enhanced through replication with additional participants.[8] SCEDs are standard in psychology and common in education, social work, and communication disorder research but can be encountered in many biomedical specialties.

Randomized and non-randomized designs

We follow the example of Cochrane[9] and others[10] and avoid distinctions between experimental *versus* observational in favor of randomized or non-randomized. Randomized trials are relatively less variable compared to non-randomized studies. The research question in randomized trials must be specific. It is investigated by comparison of intervention and control groups that should be homogeneous as well as randomly assigned. When possible, blinding of patients, interventionists, and assessors is recommended. Randomized trials are typically used to test hypotheses about new or untested interventions.

In contrast, NRSI represent a number of diverse designs that are commonly classified using ambiguous labels (Table SF2B).

Table SF2B: Common labels for non-randomized studies of interventions[a,b]

Non-randomized controlled trial
Controlled before-and-after study
Controlled interrupted time series study
Interrupted time series study
Prospective cohort study
Retrospective cohort study
Historically controlled study
Nested case–control study
Case–control study
Cross-sectional study
Before-after study

[a]Adapted from Reeves and colleagues[11]
[b]Use of these labels by systematic review authors is discouraged by Cochrane[9]

Studies that do not randomize subjects provide descriptive information (prevalence and incidence) and/or analyses of associations. Some describe a single cohort with an “exposure” (risk factor or intervention) that allows calculation of an absolute risk of a disease or disease-related outcome. More commonly, non-randomized studies compare outcomes of cohorts with different exposures that allow calculation of relative effect measures.[12,13]

Case reports and series

Case reports and series are a unique class of primary research that provide non-comparative clinical observations. They document the clinical course of one person or a small number of individuals who share some common feature.[14] This may relate to their: 1) presentation (eg, signs and symptoms of a rare disease); 2) management (eg, a specific novel treatment); 3) outcome (eg, an unusual response to or harm from a treatment); or 4) a combination of these features (eg, a rare disease treated with an uncommon procedure, an idiosyncratic adverse event after a treatment).[15] While not data driven, these studies may report clinically relevant findings that can be either quantitative and/or qualitative, and collected either prospectively or retrospectively.[16] Although often referred to as “descriptive” studies, case reports and series, unlike NRSI, cannot provide descriptive estimates of incidence and prevalence or associations.[12] However, some case series, especially those with prospective features, are often difficult to distinguish from a single-arm uncontrolled cohort study.[14,15,17] This poses a challenge for evidence synthesis authors who include case series as evidence (refer to Section 2.2 in the main text for discussion of this issue).

REFERENCES

1. Tufanaru C, Munn Z, Aromartaris E, Campbell J, Hopp L. Chapter 3: Systematic reviews of effectiveness. In: Aromartaris E, Munn Z, editors. JBI Manual for Evidence Synthesis [internet]. JBI; 2020 [cited 2022 Jan 25]. Available from: https://synthesismanual.jbi.global.
2. Lockwood C, Porritt K, Munn Z, Rittenmeyer L, Salmond S, Bjerrum M, et al. Chapter 2: Systematic reviews of qualitative evidence. In: Aromataris E, Munn Z, editors. JBI Manual for Evidence Synthesis. JBI; 2020 [cited 2022 Jul 11]. Available from: https://synthesismanual.jbi.global.
3. Ring N, Ritchie K, Mandava L JR. A guide to synthesising qualitative research for researchers undertaking health technology assessments and systematic reviews. NHS Quality Improvement Scotland; 2011 [cited 2022 Jul 12]. Available from: https://www.storre.stir.ac.uk/bitstream/1893/3205/1/HTA_MethodsofSynthesisingQualitativeLiterature_DEC101.pdf
4. Jhangiani R, Cuttler C, Leighton D. Single subject research. In: Jhangiani R, Cuttler C, Leighton D, editors. Research methods in psychology, 4th edn. [internet]. Pressbooks KPU; 2019 [cited 2022 Aug 15]. Available from: https://kpu.pressbooks.pub/psychmethods4e/part/single-subject-research/.
5. Bhandari P. Within-subjects design. Explanation, approaches, examples. Scribbr; 2022 [cited 2022 Mar 12]. https://www.scribbr.com/methodology/within-subjects-design/.
6. Wambaugh J, Schlosser R. Single-subject experimental design: an overview. CREd Library; 2014 [cited 2022 Jun 24]. Available from: https://academy.pubs.asha.org/2014/12/single-subject-experimental-design-an-overview/.
7. Janosky J, Leininger S, Hoerger M, Libkuman T. Methodological framework for single subject designs. In: Janosky J, Leininger S, Hoerger M, Libkuman T, editors. Single subject designs in biomedicine. Springer; 2009.
8. Horner R, Scott A. Single-subject design. In: Salkind NJ, editor. Encyclopedia of research design. Sage Publications; 2012.
9. Reeves BC, Deeks JJ, Higgins JPT, Shea B, Tugwell P, Wells GA. Including non-randomized studies on intervention effects. In: Higgins J, Thomas J, Chandler J, Cumpston M, Li T, MJ P,

et al., editors. Cochrane handbook for systematic reviews of interventions. Cochrane; 2022 [cited 2022 Mar 1]. Available from: https://training.cochrane.org/handbook/current/chapter-24.

10. Ioannidis JPA, Haidich AB, Pappa M, Pantazis N, Kokori SI, Tektonidou MG, et al. Comparison of evidence of treatment effects in randomized and nonrandomized studies. J Am Med Assoc. 2001;286(7):821–30.
11. Reeves BC, Wells GA, Waddington H. Quasi-experimental study designs series—paper 5: a checklist for classifying studies evaluating the effects on health interventions—a taxonomy without labels. J Clin Epidemiol. 2017;89:30–42.
12. Sargeant JM, O'Connor AM, Cullen JN, Makielski KM, Jones-Bitton A. What's in a name? The incorrect use of case series as a study design label in studies involving dogs and cats. J Vet Intern Med. 2017;31(4):1035–42.
13. Esene IN, Ngu J, El Zoghby M, Solaroglu I, Sikod AM, Kotb A, et al. Case series and descriptive cohort studies in neurosurgery: the confusion and solution. Child's Nerv Syst. 2014;30(8):1321–32.
14. Murad MH, Sultan S, Haffar S, Bazerbachi F. Methodological quality and synthesis of case series and case reports. Evid Based Med. 2018;23(2):60–3.
15. Dekkers O, Egger M, Altman D, Vandenbroucke JP. Distinguishing case series from cohort studies. Ann Intern Med. 2012;156(Pt 1):37–40.
16. Quintão C, Andrade P, Almeida F. How to improve the validity and reliability of a case study approach? J Interdiscip Stud Educ. 2020;9(2):273–84.
17. Mathes T, Pieper D. Clarifying the distinction between case series and cohort studies in systematic reviews of comparative studies: potential impact on body of evidence and workload. BMC Med Res Methodol. 2017;17(1):8–13.
