# Supplementary material for: Guidance to best tools and practices for systematic reviews
Source: J Pediatr Rehabil Med. 2023 Jun 7;16(2):241–73. doi: 10.3233/PRM-230019 (PMC10258887; doi:10.3233/PRM-230019)
Supplement: Supplementary File 4 [file prm-16-prm230019-s003.pdf]

Supplemental File 4: Presentation of forest plots

Forest plots display statistical results from individual studies and their meta-analyses. The phrase “forest plot” originated from the idea that the typical plot appears as a forest of lines.[1] It is important for readers (and reviewers) of systematic reviews to understand what has been done in order to interpret these statistics and graphs. This requires justifications for the statistical decisions involved (eg, choice of effect measure and statistical models used) in the methods section of a systematic review. It is equally important that key information is presented using standard formatting and clear labelling (Figure SF4-1[2]).

In a typical forest plot, tabular information about the individual studies (identity, numbers or rates of comparative groups, weighting) is included as well as details regarding their pooled analysis (overall effect estimates with confidence intervals, statistical inconsistency and heterogeneity assessments, levels of statistical significance). The results of component studies are displayed as squares centered on the point estimate of the result of each study; a horizontal line runs through the square to show its confidence interval. At the bottom, the summary estimate and its confidence interval are represented as a diamond; its center represents the pooled point estimate, and the horizontal tips its confidence interval.[1] This provides a simple visual representation of the amount of variation between the results of the studies and of the estimate of the overall result of all the studies combined.

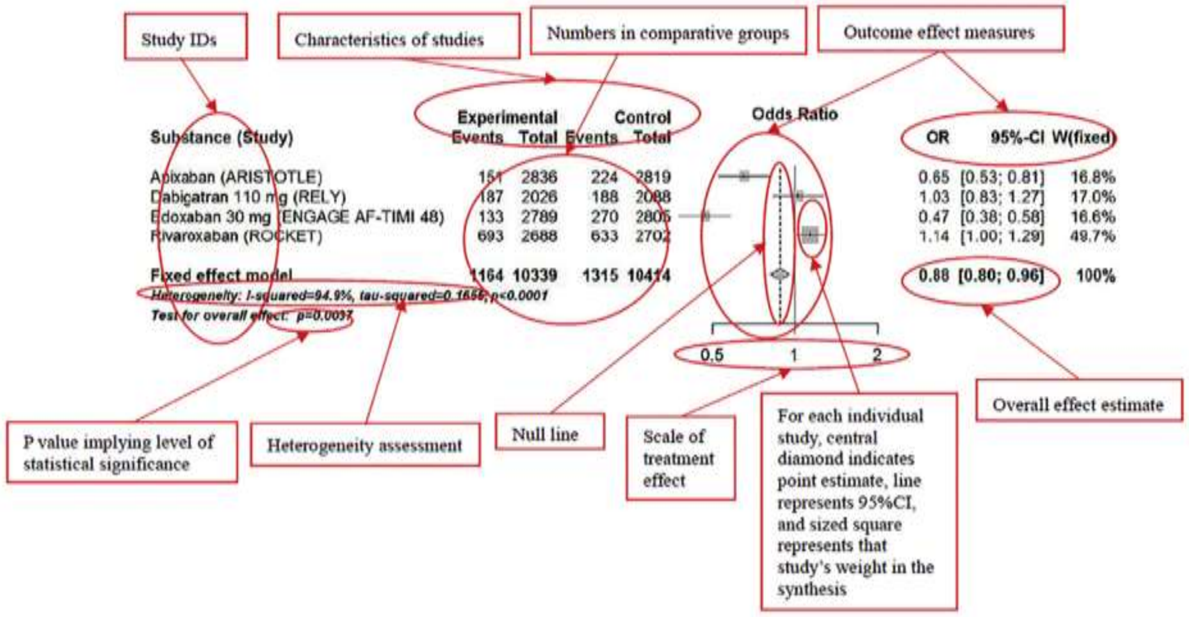

Figure SF4-1: Key information and labels to include in a forest plot. Reproduced from Li et al.[2] with permission from Elsevier.

Kolaski, K., Logan, L., & Ioannidis, J. P. A. (2023). Guidance to best tools and practices for systematic reviews. Journal of Pediatric Rehabilitation Medicine. DOI:10.3233/PRM-230019.

The addition of a vertical line on the forest plot representing a minimally important or detectable clinical difference may sometimes aid interpretation and establish if the demonstrable effect represents a meaningful difference for patients.[3,4] It is also helpful to display the details of RoB assessments for each study alongside a forest plot.[5] Cochrane suggests a standard visual aid for presentation of Cochrane RoB2 and ROBINS-I results for individual studies[6] (Figure SF4-2[7]).

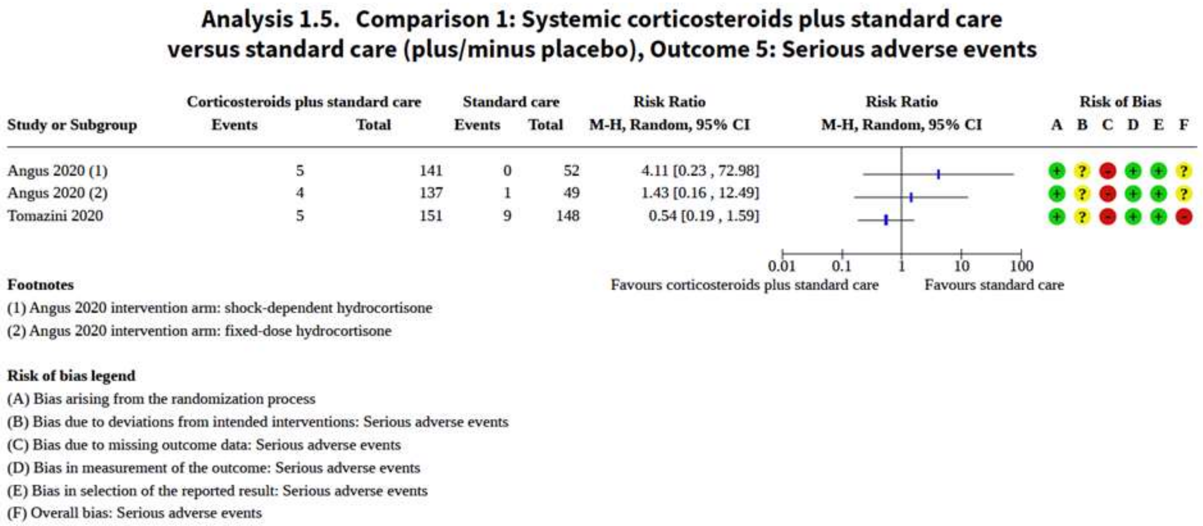

Figure SF4-2: Forest plot with presentation of RoB assessments results. Reproduced from Wagner et al.[7] with permission from John Wiley and Sons.

REFERENCES

1. Lewis S, Clarke M. Forest plots: trying to see the wood and the trees. BMJ. 2001;322(7300):1479-80.
2. Li G, Zeng J, Tian J, Levine MAH, Thabane L. Multiple uses of forest plots in presenting analysis results in health research. J Clin Epidemiol. 2020;117:89–98.
3. Kelley GA, Kelley KS, Callahan LF. Aerobic exercise and fatigue in rheumatoid arthritis participants: a meta-analysis using the minimal important difference approach. Arthritis Care Res. 2018;70(12):1735–9.
4. Zlowodzki M, Poolman RW, Kerkhoffs GM, Tornetta P, Bhandari M. How to interpret a meta-analysis and judge its value as a guide for clinical practice. Acta Orthop. 2007;78(5):598–609.
5. Johnson BT, Low RE, MacDonald H V. Panning for the gold in health research: incorporating studies' methodological quality in meta-analysis. Psychol Health. 2015;30(1):135–52.
6. Higgins J, Savovic J, Page M, Elbers R, Sterne J. Chapter 8: Assessing risk of bias in a randomized trial. In: Higgins J, Thomas J, Chandler J, Cumpston M, Li T, Page MJ, et al., editors. Cochrane handbook for systematic reviews of interventions [internet]. Cochrane; 2022 [cited 2022 Mar 20]. Available from: https://training.cochrane.org/handbook.
7. Wagner C, Griesel M, Mikolajewska A, Mueller A, Nothacker M, Kley K, et al. Systemic corticosteroids for the treatment of COVID-19. Cochrane Database Syst Rev. 2021;(8):CD014963.

Kolaski, K., Logan, L., & Ioannidis, J. P. A. (2023). Guidance to best tools and practices for systematic reviews. Journal of Pediatric Rehabilitation Medicine. DOI:10.3233/PRM-230019.

Kolaski, K., Logan, L., & Ioannidis, J. P. A. (2023). Guidance to best tools and practices for systematic reviews. Journal of Pediatric Rehabilitation Medicine. DOI:10.3233/PRM-230019.
