# Supplementary material for: Guidance to best tools and practices for systematic reviews
Source: J Pediatr Rehabil Med. 2023 Jun 7;16(2):241–73. doi: 10.3233/PRM-230019 (PMC10258887; doi:10.3233/PRM-230019)
Supplement: Supplementary File 5B [file prm-16-prm230019-s005.pdf]

Supplemental File 5B: Adaptations of GRADE for evidence syntheses

GRADE was developed to assess certainty of evidence for outcomes reported in systematic reviews of interventions; accordingly, the assessment of certainty for other types of evidence syntheses needs to consider different issues. We note developments in this area with regard to certain types of non-intervention systematic reviews; we also consider specific issues related to the adaptation of GRADE for overviews or umbrella reviews and briefly describe some examples of these advanced syntheses.

Non-intervention systematic reviews

The GRADE working group provides guidance for adapting GRADE to prognostic systematic reviews[1] and reviews of diagnostic test accuracy.[2,3] GRADE adaptations have been developed and used in systematic reviews of studies reporting on measurement properties and risk factors as well as syntheses of qualitative research.

Measurement properties

COSMIN (COnsensus-based Standards for the selection of health Measurement INstruments) uses a modified GRADE approach to assess certainty in reviews of studies reporting on measurement properties.[4] It assesses similar factors to those considered in intervention reviews (eg, risk of bias [RoB], inconsistency, indirectness, imprecision) but the starting point for downgrading or upgrading depends on the rigor of a measurement tool's development process.[5]

Qualitative

In the GRADE-CERQual (Confidence in the Evidence from Reviews of Qualitative research) approach, an overall judgment of confidence is made on the basis of an assessment of four components: methodological limitations, coherence, adequacy, and relevance.[6] In the JBI ConQual (Confidence in the Output of Qualitative research synthesis) approach, synthesized findings of a meta-aggregative review are ranked based on their creditability and dependability.[7]

Overviews or umbrella reviews

The application of the GRADE approach to overviews or umbrellas reviews requires adaptation because these evidence syntheses summarize data from systematic reviews as opposed to primary studies. It is essential that authors (and readers) understand the distinction between RoB assessments of primary studies included in a systematic review as opposed to critical appraisals of secondary studies (ie, systematic reviews) included in an overview. Another potential source of confusion relates to the GRADE ratings of the overall certainty of a body of evidence reported by individual systematic reviews included in an overview. These ratings take into account the RoB assessments of included primary studies but are not based on these exclusively; other factors are considered that may upgrade or downgrade the overall certainty rating (see Table 5.1 in the main text).

Umbrella review authors must critically appraise each included systematic review using AMSTAR-2 or ROBIS. As a separate and distinct component of overview development, authors should extract and report the GRADE assessments reported by each systematic review included as evidence. However, these may not be comparable for various reasons: in some cases, the overall certainty of evidence may not be reported; or, it may be assessed using a tool other than GRADE.[8] If the included systematic reviews in an overview do not report comparable certainty of evidence ratings, the overall certainty of evidence for the outcome of interest may need to be assessed based on the evidence at the primary study level, as opposed to the systematic review level[9]; in other words, a new systematic review is necessary. Likewise, overview authors themselves may need to determine GRADE assessments of overall certainty if they re-extract and re-analyze outcome data from systematic reviews.[8]

Proposed quantitative criteria have been applied to assess the certainty of evidence for associations of environmental risk factors with various diseases, and in genetic studies.[10-12] These criteria reflect the level of statistical support, the amount of data, the consistency across different studies, and hints of potential bias.[13] These criteria have been applied in multiple umbrella reviews summarizing risk factor associations reported by umbrella reviews and systematic reviews with and without meta-analyses.[14-16]

REFERENCES

1. Foroutan F, Guyatt G, Zuk V, Vandvik PO, Alba AC, Mustafa R, et al. GRADE Guidelines 28: Use of GRADE for the assessment of evidence about prognostic factors: rating certainty in identification of groups of patients with different absolute risks. J Clin Epidemiol. 2020;121:62–70.
2. Schünemann HJ, Mustafa RA, Brozek J, Steingart KR, Leeflang M, Murad MH, et al. GRADE guidelines: 21 part 1. Study design, risk of bias, and indirectness in rating the certainty across a body of evidence for test accuracy. J Clin Epidemiol. 2020;122:129–41.
3. Schünemann HJ, Mustafa RA, Brozek J, Steingart KR, Leeflang M, Murad MH, et al. GRADE guidelines: 21 part 2. Test accuracy: inconsistency, imprecision, publication bias, and other domains for rating the certainty of evidence and presenting it in evidence profiles and summary of findings tables. J Clin Epidemiol. 2020;122(2020):142–52.
4. Prinsen CAC, Mokkink LB, Bouter LM, Alonso J, Patrick DL, de Vet HCW, et al. COSMIN guideline for systematic reviews of patient-reported outcome measures. Qual Life Res. 2018;27(5):1147–57.
5. Mokkink LB, Prinsen CA, Patrick DL, Alonso J, Bouter LM, et al. COSMIN methodology for systematic reviews of Patient-Reported Outcome Measures (PROMs) - user manual. COSMIN; 2018 [cited 2022 Feb 15]. Available from: http://www.cosmin.nl/.
6. Lewin S, Booth A, Glenton C, Munthe-Kaas H, Rashidian A, Wainwright M, et al. Applying GRADE-CERQual to qualitative evidence synthesis findings: introduction to the series. Implement Sci. 2018;13(Suppl 1):1–10.
7. Munn Z, Porritt K, Lockwood C, Aromataris E, Pearson A. Establishing confidence in the output of qualitative research synthesis: the ConQual approach. BMC Med Res Methodol 2014;14:108.
8. Pollock M, Fernandez R, Becker LA, Pieper D, Hartling L. Chapter V: overviews of reviews. In: Higgins J, Thomas J, Chandler J, Cumpston M, Li T, Page M, et al., (Eds). *Cochrane handbook for systematic reviews of interventions* [internet]. Cochrane; 2022 [cited 2022 Mar 7]. Available from: https://training.cochrane.org/handbook/current/chapter-v.
9. Robinson KA, Chou R, Berkman ND. Integrating bodies of evidence: existing systematic reviews and primary studies. Methods guide for effectiveness and comparative effectiveness reviews . Agency for Healthcare Research and Quality; 2008.
10. Solmi M, Correll CU, Carvalho AF, Ioannidis JPA. The role of meta-analyses and umbrella reviews in assessing the harms of psychotropic medications: beyond qualitative synthesis. Epidemiol Psychiatr Sci. 2018;16:537-42.
11. Li X, Meng X, Timofeeva M, Tzoulaki I, Tsilidis KK, Ioannidis JP, et al. Serum uric acid levels and multiple health outcomes: umbrella review of evidence from observational studies, randomised controlled trials, and Mendelian randomisation studies. BMJ. 2017;357:j2376.
12. Bellou V, Belbasis L, Tzoulaki I, Evangelou E, Ioannidis JPA. Environmental risk factors and Parkinson's disease: An umbrella review of meta-analyses. Parkinsonism Relat Disord. 2016;23:1–9.
13. Janiaud P, Agarwal A. Umbrella review of umbrella reviews [internet]. OSF Registries; 2018 [cited 2022 Aug 28]. Available from: https://osf.io/g2hd7.
14. Janiaud P, Agarwal A, Tzoulaki I, Theodoratou E, Tsilidis KK, Evangelou Eet al. Validity of observational evidence on putative risk and protective factors: appraisal of 3744 meta-analyses on 57 topics. BMC Med. 2021;19(1):157.
15. Solmi M, Köhler CA, Stubbs B, Koyanagi A, Bortolato B, Monaco F, et al. Environmental risk factors and nonpharmacological and nonsurgicalinterventions for obesity: an umbrella

review of meta-analyses of cohort studies and randomized controlled trials. Eur J Clin Invest. 2018;20:e12982.

16. Belbasis L, Stefanaki I, Stratigos AJ, Evangelou E. Non-genetic risk factors for cutaneous melanoma and keratinocyte skin cancers: an umbrella review of meta-analyses. J Dermatol Sci. 2016;84(3):330–9.
